# A mathematical model for LH release in response to continuous and pulsatile exposure of gonadotrophs to GnRH

**DOI:** 10.1186/1742-4682-1-9

**Published:** 2004-09-24

**Authors:** Talitha M Washington, J Joseph Blum, Michael C Reed, P Michael Conn

**Affiliations:** 1 Department of Mathematics, College of New Rochelle, USA; 2 Department of Cell Biology, Duke University, Durham, USA; 3 Department of Mathematics, Duke University, Durham, USA; 4 Oregon National Primate Research Center, Oregon Health & Science University, Beaver-ton, USA

## Abstract

In a previous study, a model was developed to investigate the release of luteinizing hormone (LH) from pituitary cells in response to a short pulse of gonadotropin-releasing hormone (GnRH). The model included: binding of GnRH to its receptor (R), dimerization and internalization of the hormone receptor complex, interaction with a G protein, production of inositol 1,4,5-trisphosphate (IP_3_), release of calcium from the endoplasmic reticulum (ER), entrance of calcium into the cytosol via voltage gated membrane channels, pumping of calcium out of the cytosol via membrane and ER pumps, and release of LH. The extended model, presented in this paper, also includes the following physiologically important phenomena: desensitization of calcium channels; internalization of the dimerized receptors and recycling of some of the internalized receptors; an increase in G_*q *_concentration near the plasma membrane in response to receptor dimerization; and basal rates of synthesis and degradation of the receptors. With suitable choices of the parameters, good agreement with a variety of experimental data of the LH release pattern in response to pulses of various durations, repetition rates, and concentrations of GnRH were obtained. The mathematical model allows us to assess the effects of internalization and desensitization on the shapes and time courses of LH response curves.

## Background

Gonadotropin-releasing hormone (GnRH) is released by the hypothalamus in a pulsatile fashion and stimulates luteinizing hormone (LH) and follicle stimulating hormone (FSH) release by pituitary cells by a complex series of signaling processes. Although there is substantial information about various individual steps in the signaling system, there is less understanding of how these components interact to give rise to the overall behavior of the system. The frequency of pulses varies throughout the menstrual cycle increasing markedly just prior to ovulation. And, it has been observed in *in vitro *experiments on perifused pituitary cells that pulse frequency and concentration have marked (nonlinear) influences on the release of LH and FSH. The purpose of our work is to use mathematics and machine computation to understand the dynamics of this important and interesting physiological system.

In a prior study, [1], a mathematical model was developed to investigate the rate of release of luteinizing hormone from pituitary gonadotrophs in response to short pulses of gonadotropin-releasing hormone. The model included binding of the hormone to its receptor, dimerization, interaction with a G-protein, production of inositoltrisphosphate (*IP*_3_), release of calcium from the endoplasmic reticulum (ER), entrance of calcium into the cytosol via voltage gated membrane channels, pumping of calcium out of the cytosol via membrane and ER pumps, and the release of luteinizing hormone (LH). Cytosolic calcium dynamics were simplified and it was assumed that there is only one pool of releasable LH. Despite these and other simplifications, the model results matched experimental curves and enabled us to understand the reasons for the qualitative features of the LH release curves in response to GnRH pulses of short durations and different concentrations both in the presence and absence of external calcium. We note that Heinze et al, [2], created a mathematical model for LH release that reproduces some data for pulsatile administration of GnRH. Their model, however, does not include most of the important intracellular mechanisms known to play important roles; thus, they match data but do not study mechanisms. We also note that mathematical models for other aspects of the reproductive hormone system have been created: Keenan et al, [3], developed a stochastic systems model for the interactions between GnRH, LH, and testosterone; Gordan et al, [4] modelled the pulsatile release of GnRH by hypothalamic neurons.

There are four important medium-term effects that were not included in the previous study. Desensitization of the response to GnRH occurs because after GnRH binds to its receptors, some of the bound complexes are internalized and partially degraded [5]. Secondly, prolonged exposure to GnRH desensitizes the outer membrane calcium ion channels, as described in detail by Stojilkovic et al [6]. Thirdly, there exist basal rates of receptor synthesis and degradation. Finally, in response to GnRH, there also occurs an increase in the number of G_*q*/11 _proteins closely associated with the plasma membrane [7]. Incorporation of these four phenomena into the previous model allows us to analyze the contrasting effects of desensitization and signal amplification during medium-term continuous and pulsatile exposures to GnRH. We then show that the LH response curves of the enlarged model capture most of the essential features of a large number of experimental studies.

It should be noted that in the present model we ignore the long-term effects that result in changes in DNA, messenger RNA, and protein concentrations (e.g., receptor number) that are known to occur several hours after exposure to GnRH [8-11]. Thus, in the present study, we limit the time of exposure to three hours. We also ignore the long term effects of diacylglycerol which is known to cause an increase in the synthesis of LH_*α*_, the *α *subunit of the *LH *dimer [12].

## Model Development

Let *H*(*t*) represent the GnRH concentration (nM) in the surrounding medium *t *minutes after the initiation of the experiment. Initially, the hormone is bound by the receptor, R.



The bound complex HR reacts with itself to form dimers [13], denoted by HRRH.



A G_*q*/11 _protein, denoted GQ, reacts with the dimer to produce an effector, E (e.g., phospholipase C, [13]).



The values of the rate constants, *k*_1_, *k*_2_, *k*_3_, *k*_-1_, *k*_-2_, *k*_-3_, are the same as in our earlier model [1]. The abbreviations for the physiological components of the model are listed in Table 1 and all the rate constants for the current model are listed in Table 2.

**Table 1:** Glossary of Variables

H	GnRH concentration (nM)
R	Free GnRH receptor concentration (nM)
HR	Hormone-receptor complex concentration (nM)
HRRH	Hormone-receptor dimer concentration (nM)
GQ	G_*q*/11 _protein concentration (nM)
E	Effector concentration (nM)
IP_3_	Inositol 1,4,5-trisphosphate concentration (nM)
CAC	Cytosolic Ca^2+ ^concentration (*μ*M)
CAER	ER Ca^2^+ concentration (*μ*M)
CHO	Fraction of open ER Ca^2+ ^channels
LH	LH concentration (ng)

**Table 2:** Constants

R_0_	Total receptor concentration (nM)
GQ_0_	Total G_*q*/11 _protein concentration (nM)
ERUL	Resting Ca^2+ ^concentration in ER (normally 40 *μ*M)
CAE	External Ca^2+ ^concentration (normally 1000 *μ*M)
*α*	= 2 nM^-1^, see equation (17)
*β*	= 4 min^-1^, see equation (17)
*v*_1_	= 0.02 min^-1^, see equation (12)
*v*_2_	= 0.002 min^-1^, see equation (12)
*r*_0_	= 0.6, fraction internalized receptors returned
P_0_	= 8.3 × 10^-6 ^nM·min^-1^, basal rate of receptor synthesis
*γ*	= 8.3 × 10^-4 ^min^-1^, basal rate of receptor degradation
*k*_1_	= 2.5 nM^-1^·min^-1^
*k*_-1_	= 5 min^-1^
*k*_2_	= 2500 nM^-1^·min-^1^
*k*_-2_	= 5 min^-1^
*k*_3_	= 4000 nM^-1^·min^-1^
*k*_-3_	= 200 min^-1^
*k*_5_	= 2 × 10^7 ^min^-1^
*k*_-5_	= 10 min^-1^
*k*_6_	= 1 nM^-1^·min^-1^
*k*_66_	= 10 nM^-1^·min^-1^
*k*_666_	= 0
*k*_-6_	= 5 min^-1^
*k*_7_	= 2.2 *μ*M·min^-1^
*k*_8_	= 0.4 nM^-1^·min^-1^
*k*_88_	= 0
*k*_888_	= 0
*k*_9_	= 0.0002 min^-1^
*k*_10_	= 5 ng·min^-1^
*k*_11_	= 0.0008 nM-^1^·min^-1^
*k*_33_	= 2.7 min^-1^

The monomers, HR, can also interact with each other to form larger aggregates [14]. Macroaggregation and internalization occur at least 20 minutes after exposure to GnRH [14]. All of the internalized hormone and some of the receptors are then degraded, and the receptors that are not degraded are returned to the membrane [15,16]. We assume that a fraction of receptors, r_0_, can be returned intact to the membrane after a time delay of 20 minutes. Consistent with the data of [14], we assume that r_0 _= 0.6. Since we are not concerned with the details of the internalization or return processes, we adopt simple first order reactions for these processes. We assume that *n *monomers, *HR*, are internalized at a rate *k*_11 _and that *r*_0_*n *monomers that have been internalized are available to be returned to the membrane at rate *k*_11_.

There is evidence that the macroaggregates consist of an average of *n *= 100 monomers [14]. In our model, we will choose *k*_11 _= 0.08/*n *= 0.0008 nM^-1^·min^-1^. With this choice, 7% of the receptors are internalized after a 5 minute pulse of 1 nM GnRH, and 60 minutes after the initial exposure, approximately half of the internalized receptors have returned. It should be noted that it is only the combination *k*_11_*n *that occurs in the equations.

We make the following simple assumption about the recyling of receptors (consistent with the data of Maya-Nunez et al. [17] and Table 2 of Conn et al. [18]). i.e. that the formation of macroaggregates begins 20 minutes after exposure to GnRH and that the internalization and recycling process takes 20 minutes after the formation of the macroaggregates. Let *χ*(*t*) be the function that equals 1 for *t *≥ 0 and equals 0 for *t *< 0. Then, at time t, the rate of internalization of receptors is *k*_11_*n*[*HR*](*t*) and the rate of return of receptors to the membrane is *k*_11_*n*[*HR*](*t *- 40)*χ*(*t *- 40). To simplify notation, we write [*HR*]_40 _= [*HR*](*t *- 40)*χ*(*t *- 40).

Since only 60% percent of the internalized receptors are returned to the membrane after exposure to GnRH, there would not be a full recovery of receptors in the membrane. In the model we therefore include a low basal rate of receptor synthesis, *P*_0 _= 8.3 × 10^-6 ^nM·min^-1^, and degradation, *γ *= 8.3 × 10^-4 ^min^-1^. The ratio  is chosen so that the resting (in the absence of hormone) receptor concentration is *R*_0 _= 10^-2 ^nM, and the magnitude of P_0 _is chosen so that approximately  of the resting amount of receptor is produced per hour, thus ensuring a slow recovery to the steady state receptor concentration in the absence of GnRH.

The number of membrane associated GQ proteins increases in response to a GnRH agonist as described by Cornea et al [7]. For simplicity we assume that the increase of GQ proteins near the membrane depends on the concentration of HRRH in the membrane. The kinetic coefficient *k*_33 _is the parameter that determines the rate of increased concentration of GQ at the membrane in response to the formation of HRRH. We are assuming a finite pool of GQ that can be transported from the cytoplasm to the immediate vicinity of the plasma membrane. This pool is assumed to be regulated by the amount of HRRH for only the first 20 minutes, and after this time the rate of increase is negligible [7]. To fit the experimental data, we choose *k*_33 _= 2.7 min^-1 ^and multiply the kinetic coefficient *k*_33 _by *e*^-*t*/20^. With these parameters, 60 minutes after a constant exposure to 1 nM GnRH, there is a 40% increase of GQ concentration near the membrane and 120 minutes after exposure to the hormone, there is only a 43% increase. The following differential equations reflect the physiological assumptions that we have so far discussed.











We further assume that the production of IP_3 _is proportional to the concentration of E and that it is converted to inactive metabolites at a rate proportional to its concentration.



As in [1], the fraction of open channels in the ER, denoted by CHO, depends on IP_3 _concentration. CHO reaches its maximum 0.25 min after exposure to GnRH and the maximum value of CHO is 0.6. To incorporate multiple pulses, we modify the function CHO from the previous model so that it reaches its maximum 0.25 min after the start of each pulse. Thus we have



where *t*_*p *_is the time after the start of each individual pulse and, as in [1],, *α *= 2 nM^-1 ^and *β *= 4 min^-1^.

In response to GnRH, calcium is released from the ER into the cytoplasm with a rate constant ERR and is pumped back into the ER. As discussed in the previous model, the rate constant ERR increases proportionally to cytosolic calcium concentration, CAC, with a rate constant *k*_66 _and is inhibited at high CAC at a rate that is proportional to the square of CAC, with a rate constant *k*_666_. Just as in, [1], *k*_6 _= 1, *k*_66 _= 10, and *k*_666 _= 0, i.e., we ignore the inhibitory effects of calcium on reuptake of calcium into the ER.

ERR = *k*_6 _+ *k*_66_[CAC] - *k*_666_[CAC]^2 ^    (8)

The change in cytosolic calcium concentration, CAER, is then determined by the rate constant ERR, which is the rate of extrusion, multiplied by the fraction of open channels, CHO, and the difference in concentration between the calcium concentration in the cytoplasm and the endoplasmic reticulum. As in Blum et al. [1], calcium is actively transported back into the ER by pumps with the rate constant *k*_-6 _= 5 min^-1^.



As in the previous model, the volume of the ER is assumed to be 1/20 of the volume of the cytosol. CAC is determined by the rate of calcium efflux through ion channels in the ER membrane minus the rate at which calcium is being pumped back into the ER, plus the rate of calcium entry from the plasma membrane. The function VSR denotes the rate of calcium influx from extracellular calcium into the cytosol and depends on E with rate constant *k*_8 _[19] and on CAC with rate constants *k*_88 _for the influx at low CAC and *k*_888 _for the inhibitory effects at high CAC. There is considerable evidence that desensitization occurs, i.e., the fraction of open calcium channels in the cell membrane decreases soon after exposure to GnRH [18]. Since the precise mechanism of desensitization in unknown, we assume that VSR depends on E and CAC, and that channels slowly become inactive in response to exposure to GnRH, consistent with the experimental data [18]. We further assume that the fraction of open calcium channels in the outer membrane, denoted by VSRO(*t*), decreases at a linear rate of *v*_1 _= 0.02 min^-1 ^when the hormone is applied and has a minimum value of 0. In the absence of hormone, the fraction of open channels increases at a linear rate of *v*_2 _= 0.002 min^-1 ^and has a maximum value of 1. Thus, immediately a five minute pulse of 5 nM GnRH, 10% of the channels are in the refractory state and 50 minutes after the removal of the GnRH, all of the channels have recovered, consistent with experimental data; see [18] for more details. Incorporating calcium influx, pumps and leakage into the cytoplasm from the medium (the term *k*_9 _[CAE], we have



where

VSR(*t*) = (*k*_8_E(*t*) + *k*_88_[CAC](*t*) - *k*_888_([CAC])(*t*))^2^) × VSRO(*t*)     (11)

and VSRO satisfies the following.



0 ≤ VSRO(*t*) ≤ 1     (13)

Finally, the rate of release of LH depends on cytosolic calcium concentration (see Blum et al. [1] for details). Although there is evidence that there are three pools of LH in gonadotrophs, one pool, comprising of only 2% of the total LH, is released within one minute after exposure to GnRH, and the third pool is not released during continuous exposure to GnRH (Naor et al.,[20]). Therefore, as in the previous model [1], we treat LH as being released from a single pool.



The mathematical model consists of equations (1) – (14). These non-linear equations cannot be solved analytically but solutions can be obtained by machine computation. To do this, we used the solver ODE45 in Matlab.

The values of the rate constants are given in Table 2. The values for many of them are discussed in detail, with references, in our original paper, [1]. The values of the rate constants for the signalling mechanisms introduced in this paper were discussed (above) as the mechanisms were introduced. In some cases the rate constants were taken from experimental data (references given) and in other cases, where direct experimental data does not yet exist, we explained the rationale for our choices. Since the resulting model captures and explains many experimental studies (see below), these choices provide useful predictions for future experimental studies.

## Results

**Figure 1:**
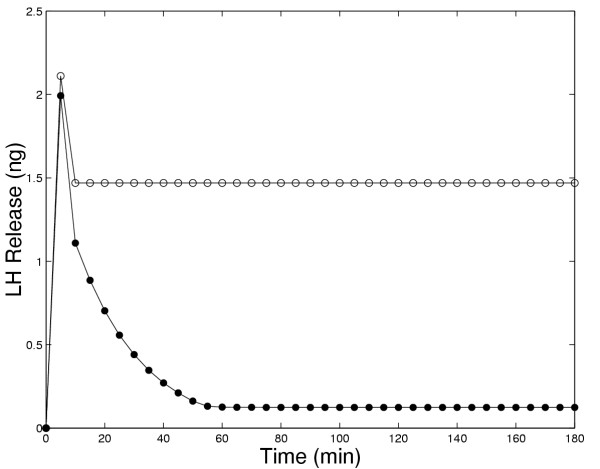
Amount of LH released in five minute intervals in response to constant exposure to 5 nM GnRH. The solid circles show the results of the present model while the open circles show the results of the original model [1]. The decay of LH release to zero is in accord with experimental results (see discussion in text); thus, new mechanisms included in the present model allow one to match this data (and other data, see other figures) from several laboratories for medium-term GnRH exposure experiments.

**Figure 4:**
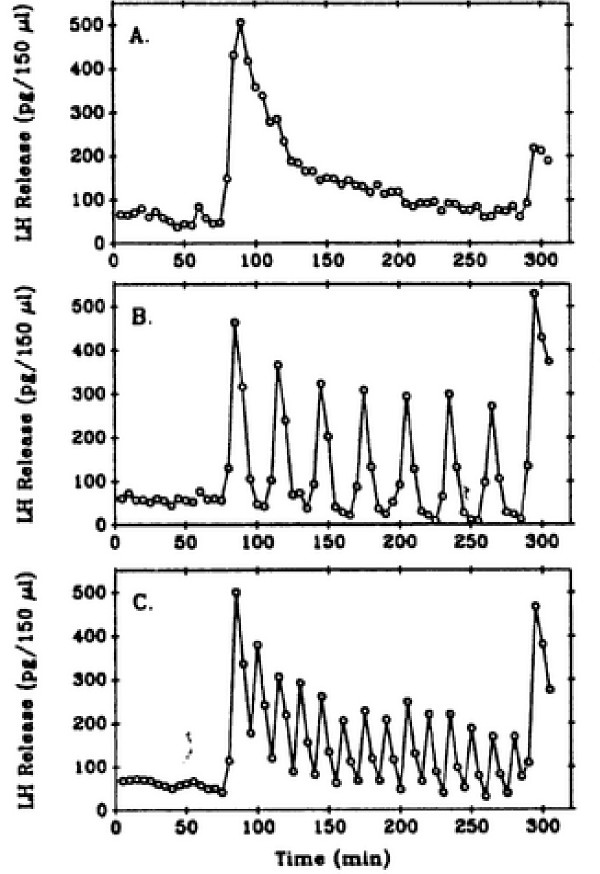
Experimental data of Hawes et al.[21]. Gonadotrophs were treated continuously with lO nM GnRH (Panel A), with 5 minute pulses every 30 minutes (Panel B), or every 15 minutes (Panel C).

In Figure 1, we compare the amounts of LH released in 5 minute intervals in the original model and the present model in response to continuous administration of 5 nM GnRH. In both models there is an initial large pulse of LH released. However, in the original model (open circles in Panel A) the long-term release plateaus at a high level, while in the present model (solid circles) the long term release declines to a low level. Panel A in Figure 4 contains experimental results of Hawes et al. [21], that clearly show show a decline in LH release to a low level after approximately 1.5 to 2 hours. Similar experimental results were obtained by Baird et al, [[22], Figure 4] and by Janovick and Conn, [[5], Figure 1, Panel A].

**Figure 2:**
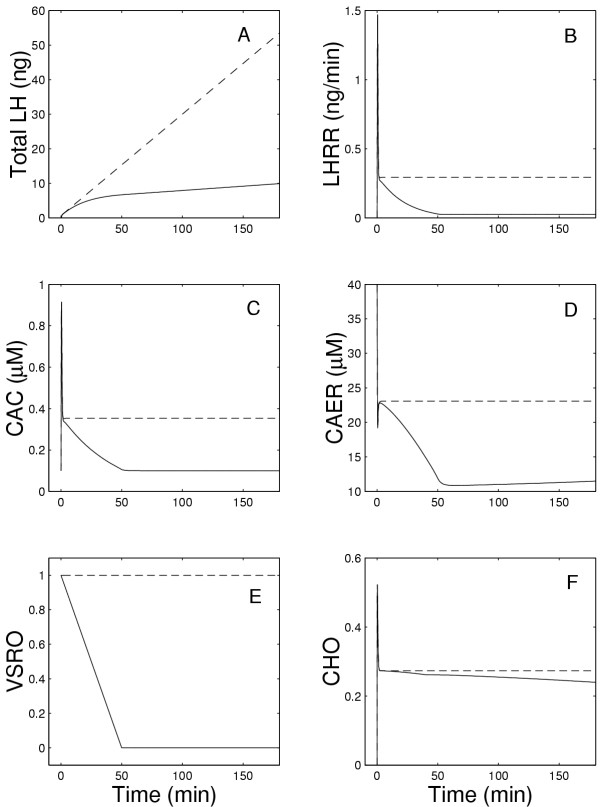
Panel A shows the total amount of LH released as a function of time during continuous exposure to 5 nM GnRH, while Panel B shows the instantaneous rate of LH release at each moment of time. Panels C and D show the calcium concentration in the cytosol and the endoplasmic reticulum, respectively. Panels E and F show the fraction of open calcium channels in the outer membrane and the endoplasmic reticulum, respectively. The solid lines show the results of the present model while the dashed lines show the results of the earlier model [1].

**Figure 3:**
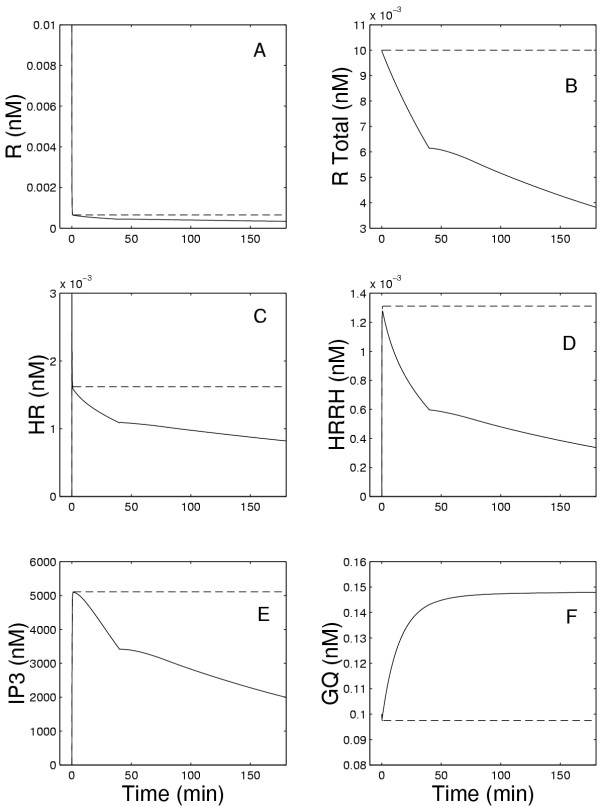
Panels A, C, and D show the concentrations of free, bound, and dimerized receptors, respectively, while Panel B shows the total amount of receptors in the membrane. Panel E shows the concentration of IP3. Panel F shows the GQ concentration at the membrane as a function of time during the continuous exposure to 5 nM GnRH. The solid lines show the results of the current model and the dashed lines show the results of the earlier model in [1].

Figures 2 and 3 show in detail the changes that occur in all components of the system during the model experiments described above. Fig. 2A shows the total amount of the LH released as a function of time while Fig. 2B shows the LH release rate (LHRR), which peaks within one minute after exposure to GnRH and then declines slowly for the next 50 minutes to a very low value in the present model. Note that LHRR is the instantaneous rate of LH release (in ng/min) while LH release in Figure 1A is in ng released in each five minute interval. In the previous model(dashed lines), LHRR plateaus at a high level (Figure 2B), so the total LH released increases linearly (Figure 2A). In the present model (solid lines), LHRR declines to a low level. In both the previous and present models, there is a rapid extrusion of calcium from the ER (Fig. 2D) and an initial rapid increase in CAC (Fig. 2C), which correlate well with the time course of the rate of change of LHRR (Figure 2B). However, the long-term behavior is different in the two models because in the present model CAC declines to a low plateau. This explains the similar drop in LH release since the rate of LH release depends on CAC (see equation (14)). The drop in CAC is caused by the desensitization of the outer membrane channels; Figure 2E shows that the fraction of open channels declines linearly to zero in 50 minutes. In the ER membrane, there is an almost instantaneous increase of open calcium channels followed by a rapid decrease and then a slight further decline (Fig. 2F).

Figures 3A and 3C show the concentrations of free receptors and receptors bound to the hormone. It can be seen that, initially in both the present and previous models, there is a very rapid decline in free receptors, R, and a very rapid increase of receptors to which GnRH has bound (HR) but have not yet dimerized. This is immediately followed, as shown in Figure 3D, by the formation of the dimers (HRRH). After this initial reaction, the concentrations of HR and HRRH remain constant in the previous model, but decline in the present model due to internalization and degradation. The recycling of receptors was assumed to start at 40 minutes (see equation (1)), which is why the rates of decrease of HR and HRRH decline at that time. Because of degradation, only a fraction (*r*_0 _= 0.6) of the internalized receptors are returned to the membrane. Thus, in the presence of continuous exposure to GnRH, the total number of receptors in the membrane continues to decline as shown in Figure 3B. The rate of change of IP3 (Fig. 3E) is closely related to the rate of change of HRRH as shown in Fig. 3D. Finally, Fig. 3F shows that there is a slow increase of approximately 43% of the concentration GQ associated with the membrane during the exposure.

**Figure 5:**
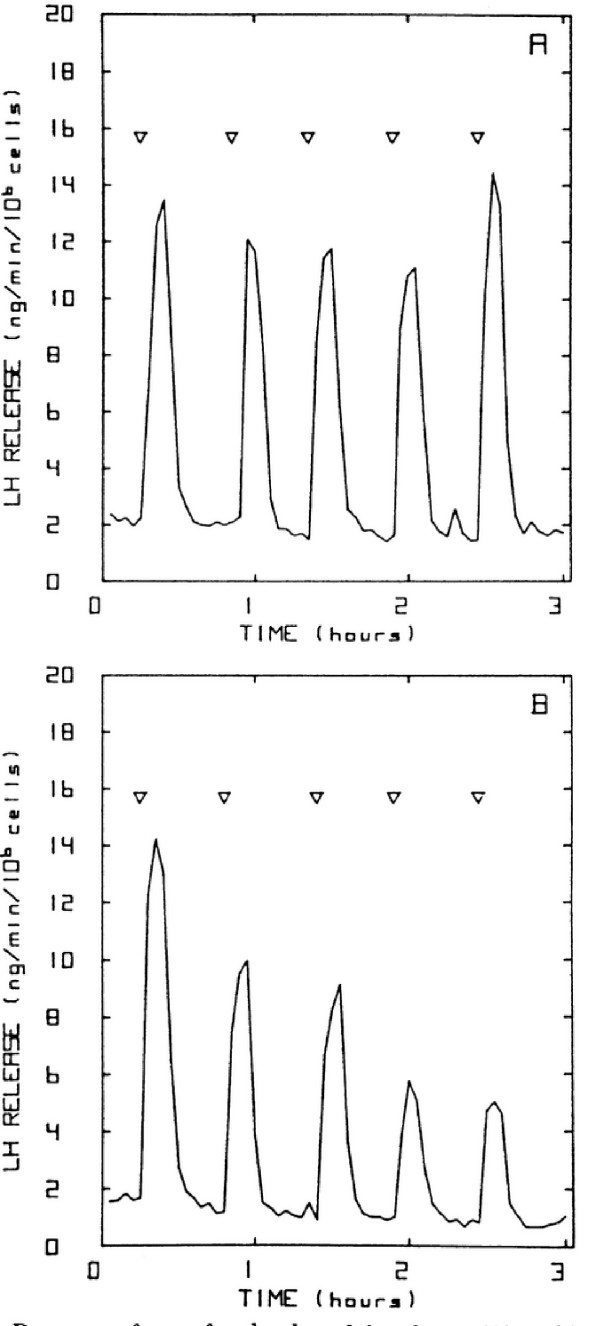
Experimental data of Baird et al.[22]. Panels A and B show the response of pubertal rat and hamster anterior pituitary cells, respectively, to six minute pulses of 10 nM GnRH.

**Figure 6:**
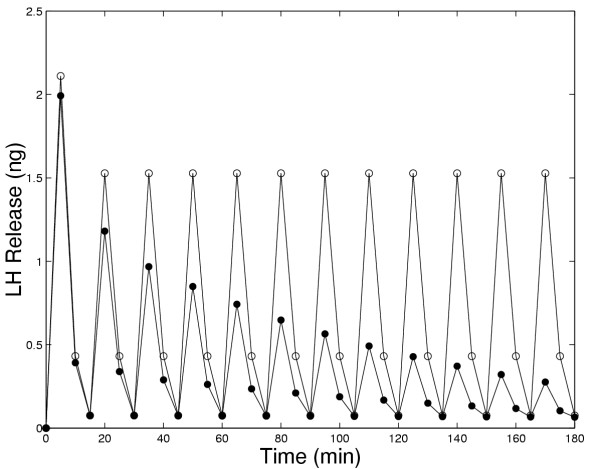
Amount of LH released as a function of time during a series of 5 minute pulses of 5 nM GnRH every 15 minutes. Open circles are the original model results and solid circles are the current model results.

**Figure 7:**
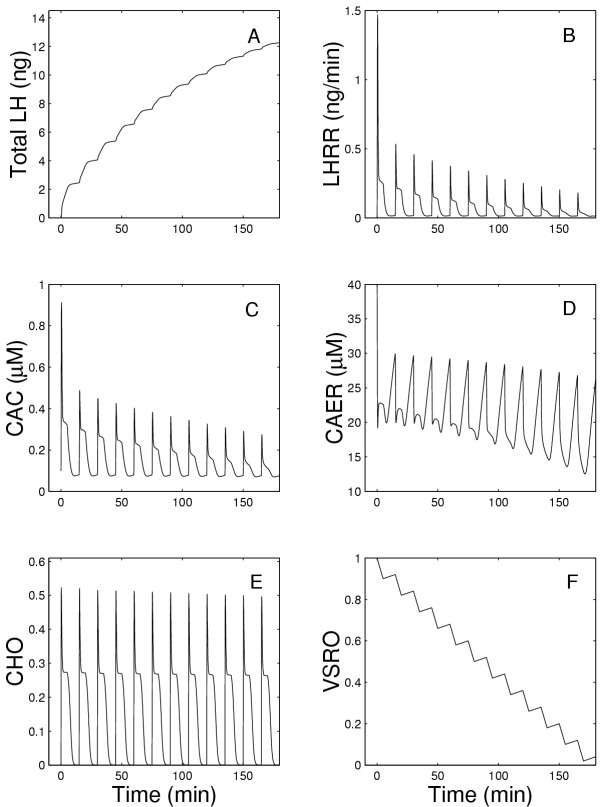
Model responses to a series of 5 minute pulses of 5 nM GnRH every 15 minutes.

**Figure 8:**
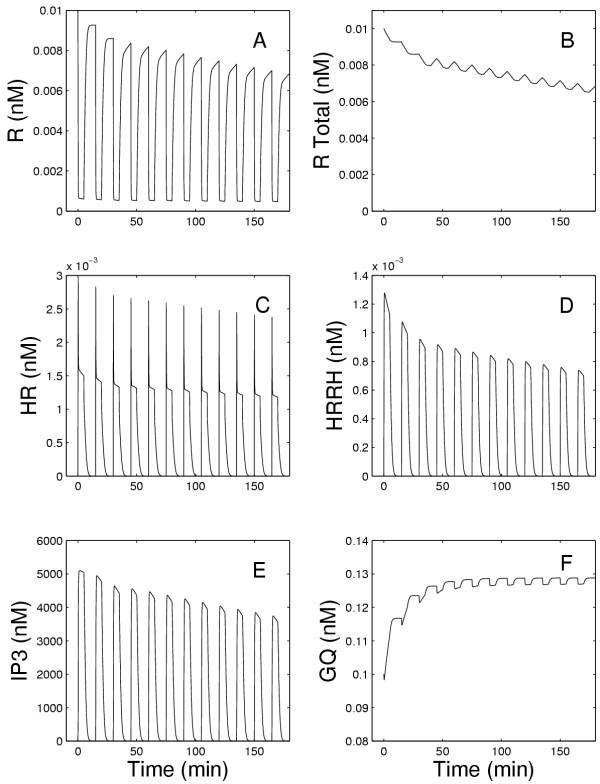
Model responses to a series of 5 minute pulses of 5 nM GnRH every 15 minutes.

Figures 6, 7, and 8 show model results for gonadotrophs exposed to 5 minute pulses of 5 nM GnRH administered every 15 minutes for a total duration of 3 hours. In the previous model (Figure 6A, open circles), there was a drop in LH release between the first and second pulse, but the same amount of LH was released in response to all subsequent pulses, contrary to experimental observations. The initial drop occurs because there is insufficient time for the calcium in the ER to refill completely (data not shown). In the present model, in response to the first pulse there is a large release of LH. In response to the second pulse considerably less LH is released, and in subsequent pulses there is a steady decline in the amount of LH released. This continual decline in LH release has been observed in a large number of experiments. Panels B and C of Figure 4 show the results of Hawes et al [21] obtained from female weanling rats. Figure 5 shows the results of experiments by Baird et al. [22] in which LH release was measured in response to similar GnRH pulse patterns in pubertal female rats (Panel A) and hamsters (Panel B). See also Janovick & Conn, [[5], Figure 1B]. This decline in the amount of LH release results both from desensitization of the calcium channels in the outer membrane and internalization of the receptors into the lysosomes, as we will see below.

The previous model (Blum et al, [1]) was intended to explain the short term response of gonadotrophs to GnRH. The success of the previous model in the first few minutes is not visible in Figures 1, 2, 3, and 6 because the long time scale compresses the first five minutes. The present model, which includes the four important medium-term processes discussed in the Introduction, now enables us to study the effects of these intracellular processes on medium-term responses, including the responses to pulses of GnRH. From now on, when we refer to the "model", we mean the present expanded model.

As shown in Figure 7B, the LH release rate decreases appreciably after the first pulse, and then continues to decrease slowly with each subsequent pulse. This arises (see equation (14)) because of the decline in the size of the cytosolic calcium pulse after each GnRH pulse as shown in Figure 7C. The ER is able to refill its calcium store to almost the same level as the preceding pulse, although the amount remaining in the ER after each pulse decreases appreciably (Figure 7D). Notice that the fraction of open channels in the outer membrane (Figure 7F) declines dramatically, while the fraction of open ER channels declines only slightly with each pulse (Figure 7E). This suggests that the primary cause of decline in the amount LH release with each GnRH pulse is the desensitization of the outer membrane. We examine this hypothesis further below.

To understand why the number of open ER channels does not decrease markedly from pulse to pulse, we refer to Figure 8. Note that the total number of receptors (Figure 8B) declines steadily by approximately 1/3 in the course of the experiment as does the number of free receptors (Figure 8A). The decline in the HRRH peaks is much greater (approximtely 40%, Figure 8D) because the formation of these dimers depends on the square of *[HR]. *However, the decline in the effector, E, which leads to the formation of IP3 (see equation (6)) is only 25% (data not shown) because of the substantial, rapid rise in GQ (Figure 8F) in response to the first pulse of GnRH. Thus, the IP3 peaks decline only about 25% (Figure 8E). Because of the Michaelis-Menten kinetics of the interaction between IP3 and the ER channels, there is an even smaller change in the fraction of open ER channels (CHO) in response to each GnRH pulse. This explains why the internalization and degradation of receptors does not have a more profound effect.

**Figure 9:**
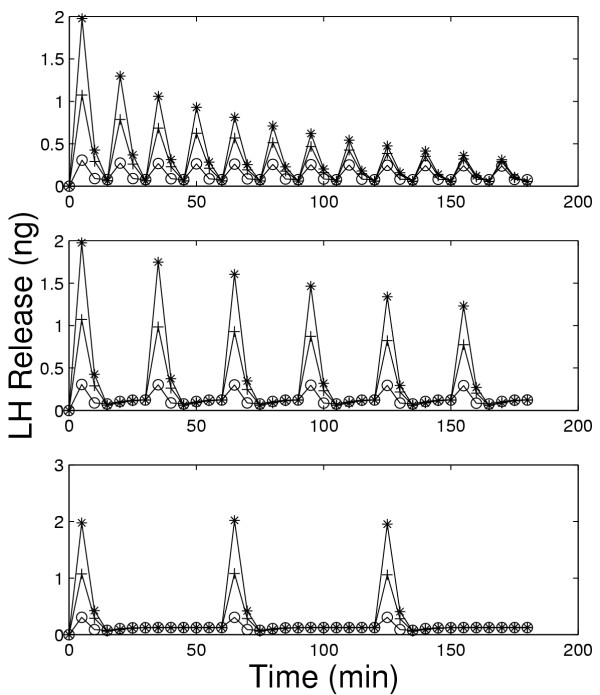
Dependence of desensitization on GnRH concentration and pulse frequency. Panels A, B, and C show model LH outputs in response to 5 minutes pulses of GnRH at pulse periods of 15 (Panel A), 30 (Panel B), and 60(Panel C) minutes. Each panel shows responses to 10 nM(*), 1 nM(+), and O.1 nM(○) GnRH.

We now investigate how the desensitization depends on pulse frequency and GnRH concentration. In Figure 4, we examined the response of the cells to pulsatile administration of a intermediate concentration of GnRH. We now examine the LH release pattern in response to pulsatile exposure to lower (0.1 nM) and higher (10 nM) concentrations of GnRH. Panels A, B, and C of Figure 9 show the model results for five minute pulses of GnRH administered every 15, 30, and 60 minutes, respectively. On each panel, the three curves correspond to pulse concentrations of 10(stars), 1 (crosses), and 0.1 (open circles) nM of GnRH, respectively. At the lowest concentration in each case there is little or no desensitization throughtout the three hour time period. At the high concentration, there is a large release of LH in response to the first pulse. For pulse period of 15 minutes, there is a large decline in the amount of LH released with each subsequent pulse (Panel A).

The decline is much smaller for pulse period of 30 minutes (Panel B). For a pulse period of 1 hour, the same amount of LH is released in response to each pulse for each GnRH concentration (Panel C). In vivo, one would not expect desensitization, so this result is consistent with experimental observations that LH pulses of the same magnitude occur approximately once an hour except just prior to ovulation (Kaiser et al,[23]). Note also that at the medium concentration of 1nM there is less desensitization at both period 15 and period 30 minutes than at the high concentration. These results are consistent with the experimental results seen by Hawes et al, [21] (our Figure 4) and Baird et al., [22] (our Figure 5), and Janovick & Conn, [[5], see their Figures 1,2,3,4].

**Figure 10:**
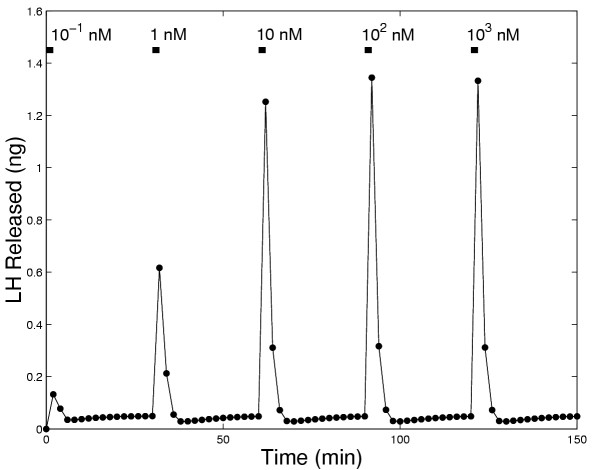
LH released during 2 minute pulses of GnRH administered every 30 minutes at the indicated increasing concentrations of GnRH.

**Figure 11:**
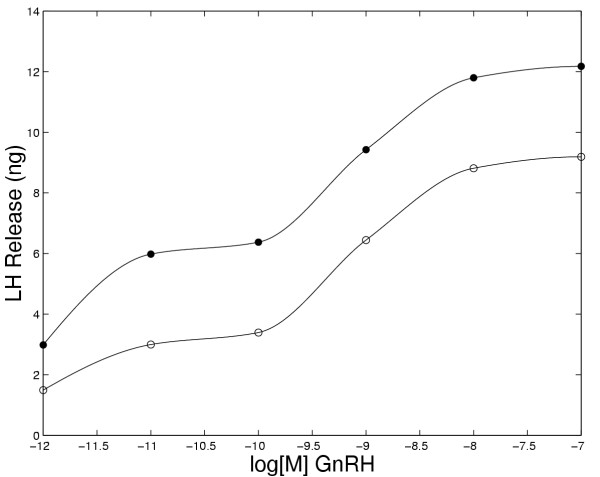
Total LH released after a 1 hour (open circles) and 2 hour (closed circles) continuous exposure to the concentrations of GnRH shown on the abscissa.

Experiments have been performed to examine LH release in response to different concentrations of GnRH. King et. al. [24] performed an experiment in which they exposed pituitary cells to increasing concentrations of GnRH for 2 minutes at 30 minute intervals for a total time of three hours. Fig. 10 shows the model results for such an experiment. The pattern of LH release by the model closely coincides with the experimental results except that at 150 minutes the model predicts a somewhat larger LH release than observed experimentally. In Figure 11 we show the total amount of LH released in the model during a one hour and a two hour exposure to increasing concentrations of GnRH. The saturating shape of each curve is sigmoidal at medium and high GnRH concentrations, as observed experimentally (see: Keri et al. [[25], Figure 1]; King et al., [[24], Figure 4]; Conn et al, [[18], Figure 6]; and Stoljikovic et al. [[26], Figure 7]). Note that, because of desensitization, the amount of LH released in 2 hours is much less than twice the amount released in one hour.

**Figure 12:**
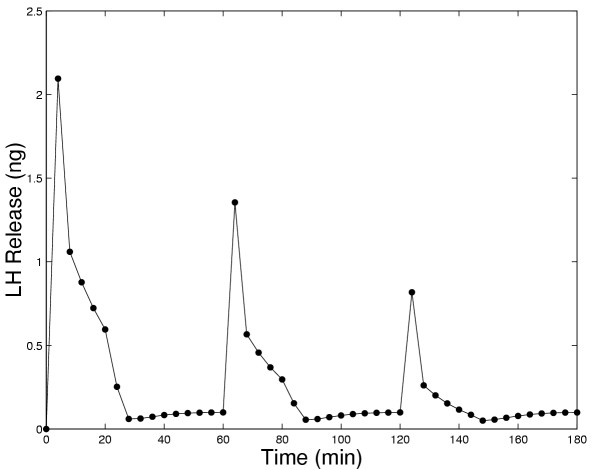
LH released in the model in response to 20 minute pulses of 100 nM GnRH administered every hour.

King et al. [24] also performed an experiment in which the cells were exposed to 20-minute pulses of 100 nM GnRH at 1-hour intervals. The model results (Figure 12) show a peak followed by a rapid decline to approximately half of the peak value and then a slower decrease to a lower level of LH release. The pattern is repeated at a reduced peak level with subsequent pulses. This pattern resembles Fig. 9 in King et al. [24] except that the experimental results show a flattening of the LH release curve late in the pulse, while the model results show a continual slow decline. Notice that both the model and experimental results show that even at one hour intervals pulses can cause desensitization if the pulse length is long enough or the frequency is high enough.

**Figure 13:**
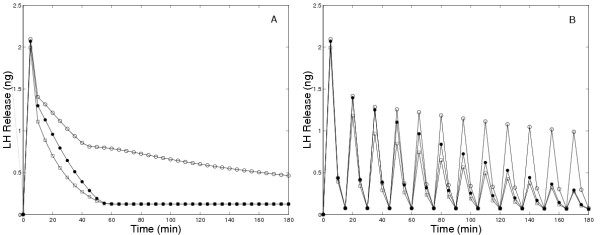
LH released during constant exposure (Panel A) and to 5 minute pulses every 15 minutes (Panel B) to 5 nM GnRH. Open circles indicate the model with no desensiti-zation of calcium channels in outer membrane (*v*_1 _= 0 and *v*_2 _= 0); solid circles indicate the model with calcium channel desensitization but with no internalization of receptors (*k*_11 _= 0); open squares indicate the full model.

To investigate which of the two desensitization mechanisms, receptor interalization or outer membrane calcium channel desensitization, plays the major role in LH release densensitization, we set either the receptor internalization to zero (i.e *k*_11 _= 0) or calcium channel densensitization to zero (*v*_0 _= 0 = *v*_1_) and compared the results to the full model for continuous and pulsatile exposures. In the full model, in response to continuous exposure there is initial rapid increase in LH release followed by a decrease to basal levels at about 40 minutes (Figure 13, Panel A, open squares), comparable to the results observed by Janovick & Conn [5]. An almost identical response occurs if *k*_11 _= 0, except that the rate of decline after the initial spike is somewhat slower(Figure 13, Panel A, solid circles). If, however, *v*_0 _= 0 and *v*_1 _= 0, while *k*_11 _retains its normal value, then the amount of LH released declines much more slowly and does not reach basal levels (Figure 13, Panel A, open circles). In response to 5 minute pulses every 15 minutes (Figure 13, Panel B), again there is a relatively small effect of setting the internalization of the receptors to zero and a much larger effect of ignoring the desensitization of the calcium channels. Thus, for continuous and pulsatile exposures up to 3 hours, internalization of the receptors plays a relatively small role in the desensitization of gonadotrophs, whereas calcium channel desensitization has a much larger effect.

In all of our previous simulations, except those in Figure 13 where we compared the two mechanisms for desensitization of LH release, the parameters in the model were never varied. We now discuss two situations where the modification of parameters gives good fits to the data and possibly new insights.

**Figure 14:**
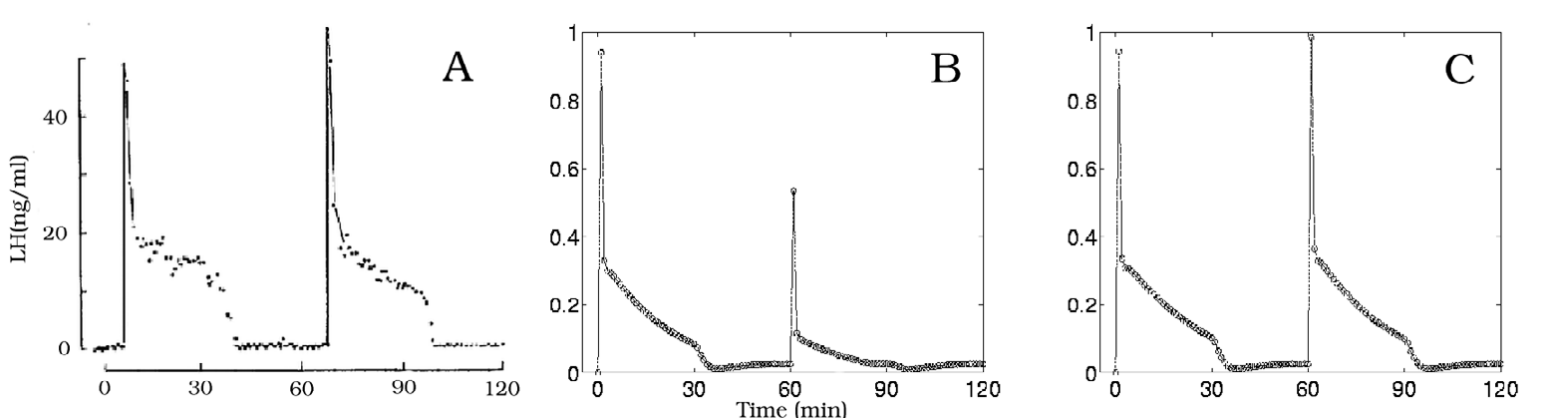
Panel A shows the results of an experiment of Stojilkovic et al.[19] in which rat pituitary cells were exposed to two 30 minute pulses of 100 nM GnRH at one hour intervals. Panel B shows the response of the present model to the same pulses. If, how-ever, the rate of recovery of the calcium channels in the outer membrane is increased from *v*_2 _= 0.002 min^-1 ^to *v*_2 _= 0.02 min^-1 ^and the rate internalization of receptors is decreased from *k*_11 _= 0.08/*n *nM^-1^·min^-1 ^to *k*_11 _= 0.04/*n *nM^-1^·min^-1^, then the present model gives responses (Panel C) similar to the exerperimental results in Panel A. The ordinate units for Panels B and C are ng.

Stojilkovic et al. [6] exposed gonadotrophs from two week old ovariectomized female rats to two 30 minute pulses of 100 nM GnRH at one hour intervals or to two 30 minute pulses of 100 nM endothelin (ET), a hormone with LH releasing activity comparable to GnRH. In response to GnRH, the peak of the response to the second pulse was actually slightly larger than the response of the first pulse (Figure 14, Panel A). However, the response to the second pulse using the present model without any change in parameters was appreciably smaller than the response to the first pulse (Figure 14, Panel B). A closer approximation to the experimental results from ovariectomized rats was obtained simply by increasing the rate of recovery of the outer membrane calcium channels from *v*_2 _= 0.002 min^-1 ^to *v*_2 _= 0.02 min^-1 ^(data not shown). If, in addition, the rate of internalization of receptors is decreased from *k*_11 _= 0.08/*n *nM^-1^·min^-1 ^to *k*_11 _= 0.04/*n *nM^-1^·min^-1^, the response to the second pulse of GnRH was very similar to that observed experimentally, as shown in Figure 14, Panel C.

**Figure 15:**
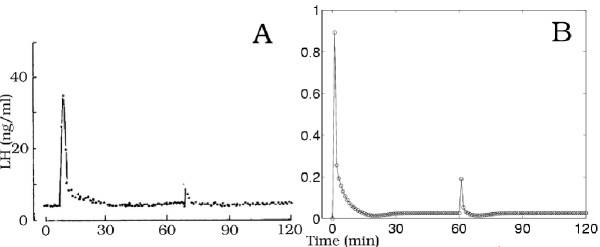
Panel A shows the results of an experiment of Stojilkovic et al. [6] in which rat pituitary cells were exposed to two 30 minute pulses of 100 nM endothelin at one hour intervals. Note that the response to Endothelin is markedly different than the response to GnRH in Panel A of Figure 14. If we change the present model by increasing in internalization of receptors (*k*_11 _= 0.8/*n *nM^-1^·min^-1^) and a decreasing the return of internalized receptors (from 60% to 10%), then the model (Panel B) closely approximates the experimental results. The ordinate units for Panels B are ng.

In the experiments of Stojilkovic et al. [6], the first 30 minute pulse of ET provokes a high peak in LH release, as for GnRH. This peak, however, is followed by a rapid decline to basal levels. Furthermore, only a very small amount of LH was released in response to the second pulse of ET (see our Figure 15, Panel A). They attributed this rapid desensitization in part to rapid internalization of the ET receptors (see also Stojilkovic et al. [27]). To test this hypothesis, we increased the rate of internalization of these receptors from *k*_11 _= 0.08/*n *to *k*_11 _= 0.8/*n*. Although this decreased the amount LH released appreciably on the second pulse, the amount of LH released was not reduced to a comparably low level as observed experimentally. We therefore also decreased the amount of return of internalized ET receptors to the membrane, r_0_, from 60% to 10%. As shown in Fig. 15, Panel B, the model now produces a good match to the experimental data. We also note that as in the experimental data, the LH released in response to ET with the receptor internalization modification returns to basal level much faster than in the case of GnRH.

A similar result can be acheived by introducing desensitization of both the outer membrane and ER calcium channels instead of changing the internalization and recycling of the receptors. The parameters for the desensitization of the calcium channels in the outer membrane were increased from 0.02 min^-1 ^to 0.4 min^-1^. This resulted in approximately 70% decrease in the magnitude of response to the second pulse of ET, but further increase in *v*_1 _did not cause any further reduction in magnitude. Since there is evidence suggesting that the calcium channels in the ER desensitize in response to GnRH (Conn et al [18]), we introduced this desensitization into the model to see if ER desensitization might also be occuring in response to ET. For simplicity, the rates of desensitization and of recovery of the ER calcium channels were chosen to be identical to that of the desensitization of the outer calcium channels. By including desensitization of both the outer membrane and ER calcium channels, the amount of LH released in response to the second pulse of ET was as small as was observed experimentally (data not shown). Thus, our current model, with few parameter changes, appears capable of explaining the responses to endothelin. However, in the absence of more detailed experimental data (for example responses to pulses of different durations and frequencies, etc.) we cannot at present distinguish between the two above proposed mechanisms.

## Discussion

We have extended our previous model to include receptor internalization and partial degradation, outer membrane calcium channel desensitization, basal levels of receptor synthesis and destruction, and an increase in the number of G_*q*/11 _proteins closely associated with the plasma membrane. With these additions we are now able to examine the behavior of the model system over medium term (up to three hours) exposures to GnRH and to a variety of pulsatile exposures. We have compared the model behavior to many such different experiments and found that it shows the essential response properties of the gonadotrophs. Furthermore, since the model includes many of the intracellualar physiological processes, we have used the model to investigate and understand the mechanisms that give rise to the various experimental results.

**Figure 16:**
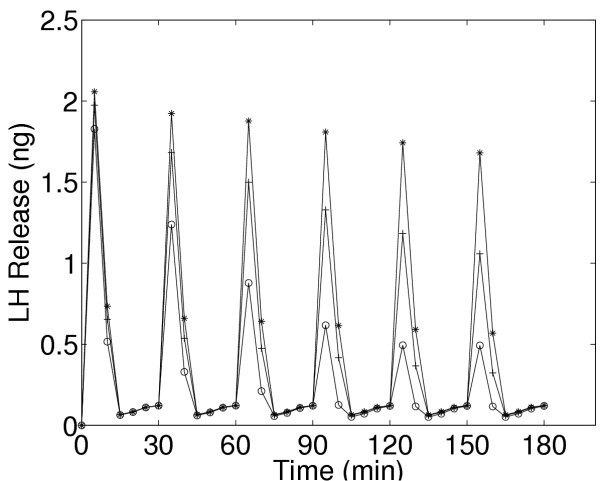
Model responses to six minutes pulses of 10 nM GnRH every 30 minutes with the standard parameters (crosses), with *v*_1 _changed from 0.02/min to 0.005/min (stars) or to 0.05/min (open circles). The weak desensitization (stars) is similar to that of the rat in the experiments of Baird et al.[22](our Figure 5A) and the strong desensitization (open circles) is similar to that seen in the hamster (our Figure 5B).

We note that the response of gonadotrophs to GnRH depends on the method of cell preparation, the stage of the estrous cycle, and the particular animals and species used. Thus, the real physiological parameters will vary in these different situations. Therefore, one would not expect that our model with the fixed set of "standard" parameters (used for the simulations in Figures 1,2,3 and 6,7,8,9,10,11,12) would match perfectly any particular set of experimental data. Of course, one can tune the model by adjusting parameters. For example, notice that the degree of desensitization to six minute pulses of 10 nM GnRH is differs markedly for the pubertal female rats and hamsters in the experiments of Baird et al. [22] as shown in Figure 5. The model behavior with standard parameters gives less desensitization than the hamster and more than the rat (see open circles in Figure 16). By changing the model parameter *v*_1 _(the rate of desensitization of the outer membrane calcium channels) from 0.02/min to 0.005/min we obtain a good match to the rat data (closed circles in Figure 16), and by changing *v*_1 _from 0.02/min to 0.05/min we obtain a good match to the hamster data (stars in Figure 16). This does not prove, of course, that it is only physiological variation in this parameter that gives the different experimental results, but it does suggest the specific experiments that could be performed to test this hypothesis.

We used parameter variation to investigate whether receptor internalization or outer membrane calcium channel desensitization plays the major role in LH release desensitization and concluded that outer membrane calcium channel desensitization is more important, at least in the experiments of Janovick and Conn [5]. We also used parameter variation to show that changing two parameters (the rate of recovery of the outer membrane channels and the rate of receptor internalization) the model gives good matches to the data of Stoljilkovic et al [19], on LH responses to pulses of endothelin. This strongly suggests that the same intracellular mechanisms are primarily responsible for the LH responses to GnRH and endothelin.

It is important to note that the model ignores a number of processes that play a role in the long-term response to GnRH. In gonadotrophs, depending on the frequency and duration of exposure to pulses of GnRH, there may be an increase or decrease in the number of receptors in the cell membrane due to changes in gene expression and/or mRNA translation [28,9,8,30]. These long-term effects are not important for the current study but will be included in future work. It is also known that there is activation of protein kinase C in gonadotrophs exposed to GnRH [20], but while PKC may not be involved in GnRH-mediated LH release [31], PKC may have other roles in the pituitary, such as to modulate gonadotroph responsiveness to GnRH [32]. Another aspect that our model ignores is the rapid calcium concentration oscillations in the cytosol. As shown by Stojilkovic and Tomic [33], the frequency of the oscillations affect the LH release. In the present model, as in the previous model [1], for simplicity we have assumed that the average cytosolic calcium concentration is an adequate approximation to the rapid oscillatory responses. Finally, we note (Stanislaus et al, [34]) that there is evidence that the GnRH receptor interacts with more than one G protein and Stanislaus et al,[13], have proposed that this underlies the differential regulation of the release of luteinizing hormone and follicle stimulating hormone. We plan to address these questions in future work.

## Authors' Contributions

Washington and Reed contributed mostly to the mathematical development, Conn contributed to the physiological analysis, and Blum contributed to both.

## Competing Interests

The authors declare that they have no competing interests.
